# Benchmarking knowledge, attitudes and practices on food allergies and celiac disease among food service staff: exploratory findings and policy gaps

**DOI:** 10.3389/fnut.2025.1644906

**Published:** 2025-10-23

**Authors:** Ximena Figueroa-Gómez, Juan Manuel Rodríguez, María-Jesús Oliveras-López, Marcelo Poyanco, Herminia López, Fernando Martínez-Martínez, Magdalena Araya

**Affiliations:** 1 Department of Pharmacy and Pharmaceutical Technology, Faculty of Pharmacy, University of Granada, Granada, Spain; 2 Human Nutrition Unit, Institute of Nutrition and Food Technology (INTA), University of Chile, Santiago, Chile; 3 Institute of Nutrition and Food Technology (INTA), University of Chile, Santiago, Chile; 4 Department of Molecular Biology and Biochemical Engineering, University Pablo de Olavide, Seville, Spain; 5 Faculty of Economic and Administrative Sciences, University of Valparaíso, Viña del Mar, Región de Valparaíso, Chile; 6 Department of Nutrition and Bromatology, Faculty of Pharmacy, University of Granada, Granada, Spain; 7 Department of Pharmacy and Pharmaceutical Technology, Faculty of Pharmacy, University of Granada, Granada, Spain

**Keywords:** food service, food allergy, celiac disease, knowledge, attitudes, practices

## Abstract

This exploratory study examines how foodservice workers in Chile manage the dietary needs of individuals with food allergies (FA) and celiac disease (CD), presenting Chile as a case study that, despite its formal classification as a high-income country, shares regulatory and operational gaps with emerging economies in transition. A cross-sectional survey of 397 restaurant and foodservice employees in Santiago evaluated their knowledge, attitudes, and practices (KAP) regarding FA and CD. Simultaneously, a structured narrative review of 26 international studies published between 2010 and 2024 was conducted to benchmark national findings against global trends. Results revealed that 87.5% of participants had never received formal training, and less than 2% achieved acceptable performance, defined as ≥50% correct responses across all KAP dimensions. Statistically significant associations were found between higher KAP scores and factors such as education level, managerial position, and length of professional experience. Conversely, foreign-born workers and those in fast-food settings showed lower performance, exposing structural vulnerabilities. The international comparison underscored widespread deficiencies even in countries with allergen regulations, highlighting that legislation alone is insufficient without mandatory training and enforcement. These findings highlight serious risks and support a phased national strategy, beginning with pilot interventions, rather than immediate policy change. This study also offers a replicable methodology for assessing and improving allergen safety in foodservice environments across emerging economies.

## 1 Introduction

The significant rise in food allergies (FA) and celiac disease (CD) over the past century is a growing public health concern, especially in foodservice settings where individuals with dietary restrictions are at increased risk. Food allergies are defined as adverse immune responses to food proteins and other components, categorized as IgE-mediated, non-IgE-mediated, or mixed (1, 2). In contrast, celiac disease is an autoimmune disorder triggered by gluten ingestion in genetically predisposed individuals, resulting in intestinal damage and various autoimmune systemic effects (3). Although FA and CD are distinct disorders, both require strict dietary avoidance; however, their management trajectories diverge. FA carries acute, life-threatening risks such as anaphylaxis, requiring emergency preparedness, whereas CD involves long-term risks from chronic gluten exposure, including autoimmune comorbidities and malignancies, demanding sustained dietary adherence (4, 5). Recognizing these differences highlights the need for foodservice training that addresses both acute emergencies (e.g., anaphylaxis in FA) and chronic risk prevention (e.g., sustained gluten avoidance in CD) (6).

Despite improvements in the availability of allergen-free and gluten-free products, dining out remains risky due to inconsistent labelling, limited staff knowledge, and persistent cross-contact during food preparation (7, 8). Research shows that even when consumers communicate their dietary needs, meals may still contain hidden allergens or gluten due to miscommunication, inadequate protocols, or lack of awareness (9). For these populations, foodservice settings represent not just a social or culinary experience but a potential health hazard.

Foodservice workers play a central role in moderating these risks. International organizations such as the WHO, Codex Alimentarius, and the FDA emphasize the need for staff to clearly communicate the ingredients and allergens used, and contamination risks (10, 11). However, multiple studies report insufficient knowledge and unsafe practices among foodservice personnel, even in countries with existing regulations (12–15). For example, studies in the United States and Canada have shown that restaurant workers often cannot identify major allergens, recognize symptoms of allergic reactions, or implement effective cross-contact prevention protocols (13, 16). In emerging economies, where enforcement mechanisms are weaker, the risks are expanded by the absence of structured training and regulatory oversight (12, 14, 17). These findings suggest that the mere existence of regulation is not sufficient; without implementation, training, and supervision, foodservice environments remain high-risk for allergic and celiac individuals.

This international variability reflects inconsistent enforcement and diverse training practices. The EU Regulation 1169/2011 (18) mandates the disclosure of 14 priority allergens in packaged and unpackaged foods. In the U. S., FALCPA and the FDA Food Code promote allergen training and menu transparency. Targeted training interventions in Lebanon and Canada have significantly improved staff KAP indicators (13, 14). In Chile, legislation (acts 20.606 and 21.362) (19, 20) only applies to packaged foods and public institutions, leaving most restaurants out of the regulatory framework. Moreover, the national sanitary regulation (RSA DS 977/96) (21) does not require allergen disclosure or specific training for foodservice personnel on handling allergens and gluten. This regulatory gap places allergic and celiac consumers at a structural disadvantage, with no institutional mechanisms to ensure meal safety outside the home.

Given this context, the present study combines two components: (1) an exploratory cross-sectional survey of foodservice workers in Santiago, Chile, to assess knowledge, attitudes, and practices (KAP) regarding FA and CD; and (2) a normative-comparative analysis of international allergen regulations and food safety protocols. These complementary components enable a comprehensive diagnosis of Chile’s foodservice vulnerabilities and allow for benchmarking against international standards. Thus, Chile is presented as a case study that, although formally classified as a high-income economy, remains the OECD member with the lowest GDP per capita and shows regulatory gaps similar to upper-middle-income contexts. By integrating empirical data with global policy comparisons, this research aims to contribute to the development of tailored regulatory strategies that enhance protection for vulnerable consumer populations.

## 2 Materials and methods

### 2.1 Study background and integration with prior research

This study constitutes the third publication in a research series focused on the challenges and risk management of special dietary needs, particularly food allergies (FA) and celiac disease (CD), within the foodservice sector. The first article in the series was a systematic review and meta-analysis examining knowledge, attitudes, and practices (KAP) among foodservice workers internationally (22). The second explored the lived experiences and perceived risks of individuals with FA and CD when dining out, using data from Chilean consumers (6). These foundational studies provided key insights and methodological precedents that informed the current research, particularly in the design of the survey instrument, the structure of KAP indicators, and the identification of critical contextual variables for foodservice environments.

Acceptable performance was operationally defined as achieving ≥50% correct responses across all three KAP dimensions (knowledge, attitudes, and practices). This threshold has been used in previous studies as a minimal benchmark for adequate competence. An exploratory 80% cutoff was also tested, but it classified nearly all respondents as insufficient; therefore, the 50% threshold was retained as the main criterion.

### 2.2 Operational procedure

This cross–sectional prospective study evaluated a convenience sample of 10 gastronomic centers in Santiago, the capital city of Chile. Locations were randomly selected following the Corporación Regional de Santiago, which provides national gastronomic tourist information. Restaurant staff, managers, cooks, and waiters were invited to participate, aiming at 30 persons per district, one or more per restaurant, to finally obtain around 300 interviews. This methodology was chosen because no prior data were available to formally calculate the sample size, and methodological literature indicates that a sample of about 300 participants is generally sufficient to achieve acceptable statistical power for detecting medium-sized effects at a standard significance level (23–25).

### 2.3 Study group and questionnaire

The literature review revealed no validated questionnaire to assess the matter of interest; therefore, an ad-hoc questionnaire was developed based on existing scientific literature and adapted by the authors. Items were selected and adapted from previously published surveys evaluating food allergy knowledge, attitudes, and practices, and reviewed by a small expert group with experience in food allergy research and management. The final version included 51 questions addressing sociodemographic information, basic knowledge about celiac disease and food allergies, the need for special diets, attitudes, practices when a customer is served, and general safety concerns when providing food service. Some questions were multiple choice, others were true/false, or followed a Likert scale. Anonymity and confidentiality were guaranteed. The questionnaire was applied during interviews conducted by one of the authors (XF) and was piloted with 37 individuals before protocol initiation. The IRB of INTA, University of Chile, approved the protocol (Document #21, June 2, 2021). The study was conducted during the Spring months, and a total of 397 individuals completed the survey, including restaurant staff, managers, cooks, and waiters. No services were excluded. Results were analyzed with descriptive statistics using the SPSS and GraphPad Prism 7 programs. For variables with extremely small subgroup sizes (e.g., only ~1% classified as ‘good’ in attitudes or practices), no inferential tests were applied. These categories were reported descriptively in terms of frequencies and percentages only.

### 2.4 Structured narrative review and comparative benchmarking

The second objective of this study was to place the national findings within a comparative international framework to better interpret the observed gaps in the management of restrictive diets in foodservice operations. To achieve this, a structured narrative review was conducted to identify trends and common patterns in the performance of foodservice workers regarding food allergies and celiac disease across different countries. The review was conducted as a structured narrative review rather than a formal systematic review; therefore, PRISMA reporting standards were not applied, and no quantitative synthesis was performed. Transparency was maintained through predefined PICO criteria, a coding matrix documenting inclusion and exclusion decisions, and independent review by two authors. The search strategy was systematic in design, drawing on the equation and descriptors reported in our prior systematic review of KAP instruments in foodservice allergen management (22). This structured process ensured rigor and traceability while recognizing the narrative character of the synthesis. Instead, the review aimed to construct a comparative matrix that could inform and support the interpretation of Chilean findings through a normative and operational benchmarking lens.

The PICO model guided the search strategy, targeting: Population (foodservice and kitchen workers), Intervention (knowledge, attitudes, and practices), and Outcomes (KAP performance according to international regulatory frameworks). Searches were performed in PubMed, Scopus, Web of Science, and Cochrane, using both MeSH terms and free-text keywords in English and Spanish. Sample search string: (“food allergy” OR “celiac disease” OR “gluten free”) AND (“restaurants” OR “hotel” OR “kitchen workers” OR “food handlers”) AND (“training” OR “awareness” OR “practice”) NOT immunotherapy.

Searches were conducted in two rounds: January 28, 2022, and January 23, 2024. A total of 26 articles were retrieved. Inclusion criteria were: Original empirical studies (quantitative or mixed-methods); Explicit evaluation of at least one KAP dimension; Focus on foodservice settings (restaurants, hotels, cafés); Articles published in English or Spanish.

Narrative reviews, book chapters, and studies limited to healthcare or school settings were excluded. Selected articles were imported into Mendeley and Zotero for reference management and manually screened by the research team. The eligible studies were then systematized in an Excel matrix that captured key dimensions: country of origin, applicable food allergen legislation (when available), and author-reported findings on knowledge, attitudes, and practices (KAP).

To enable interpretive comparisons across studies, we developed a structured synthesis based on empirical scientific literature published between 2010 and 2024. Each article was analyzed individually by examining literal statements made by the original authors in their results, discussion, or conclusions sections, specifically regarding food allergy-related knowledge (K), attitudes (A), and practices (P) among foodservice personnel.

A qualitative ordinal scale was applied to evaluate each KAP dimension using the following criteria:

- +++ (good): when the study indicated that personnel had high or adequate knowledge, showed positive attitudes, or demonstrated appropriate practices concerning food allergy or celiac disease management;
- ++ (moderate): when the findings reflected an intermediate or acceptable level, albeit with some limitations or areas for improvement;
- + (insufficient): when the article explicitly reported significant knowledge deficits, inadequate attitudes, or suboptimal or incorrect practices;
— (not stated): when no explicit information was provided for a given KAP dimension.

Importantly, this classification was based exclusively on the literal language used by the study authors, without reinterpretation or extrapolation. The goal was to ensure maximum fidelity to the original empirical data and avoid bias. Coding was conducted independently by four reviewers and consolidated through consensus. In the cases where the authors’ statements were ambiguous, classification decisions were guided by internationally recognized standards such as the Codex Alimentarius and the FAO/WHO guidelines on allergen management.

This synthesis approach adhered to the principles of directed qualitative content analysis, as outlined by Hsieh and Shannon (26). Rather than generating inductive codes, the analysis was guided by a predefined interpretive rubric informed by existing literature and regulatory frameworks. The classification was not intended to produce a grounded theory but rather to harmonize heterogeneous findings across studies while preserving their contextual integrity. Although qualitative and interpretive in nature, the matrix provided a standardized lens through which to support comparative and policy-relevant insights across international contexts.

The results of this review were synthesized in a comparative matrix (Table 1), which presents the selected studies, country of origin, relevant national legislation, literal author statements regarding KAP, and the assigned performance scores. This matrix is presented in the Results section as a methodological resource to support comparative interpretation and international benchmarking.

**Table 1:** Knowledge, attitudes, and practices of restaurant workers reported between 2010 and 2024.

Author	Country	Legislation	Authors statement		Score
North America					
Lee and Xu (2015) (27)	USA	U. S. Food and Drug Administration FDA/Food Allergen Labeling and Consumer Protection Act, FALCPA.	K:	*“This study further identified knowledge areas that need improvement.”*	+
			A:	*“There is also a need to change the attitudes of restaurateurs and how they view food allergies [...]; restaurateurs need to be more accommodating and more open-minded when allergen-free foods are requested.”*	+
			P:	*“These efforts should be continued and more restaurants should be encouraged to implement some of these practices.”*	++
Radke et al. (2016) (23)	USA		K:	*“However, we identified important gaps in knowledge [...] Another troubling finding was that more than 10% of managers and staff believe that someone with a food allergy can safely consume a small amount of that allergen.”*	+
			A:	*“Overall, these findings suggest that managers, food workers, and servers are knowledgeable and have positive attitudes about accommodating customers with food allergies.”*	+++
			P:		−
Lee and Sozen (2016) (39)	USA		K:	*“Some of the knowledge areas showing a need for improvement were consistent with results from previous research, including identification of major allergens, differentiation between food allergies and food intolerances, and handling of food allergens.”*	+
			A:	*“The results from the attitude section of the survey showed that, in general, participants were aware of the increased prevalence of food allergies and were concerned about food allergies in the restaurant industry.”*	++
			P:	*“Future training needs to emphasize information that is critical to ensure customer safety and has been shown to be lacking…”*	+
Dupuis et al. (2016) (16)	USA		K:	*“Participants revealed fundamental knowledge gaps regarding how to reduce the risk of and respond to food allergy adverse events.”*	+
			A:	*“Participants indicated a high level of willingness to assist food allergic customers and to learn more about food allergies…”*	+++	P		
			Radke et al. (2017) (40)	USA	K:	*“Restaurants should ensure that all staff members are knowledgeable about food allergies, from preventing cross-contact to knowing how to respond in an emergency.”*	+
A:					−
P:	*“Approximately half of surveyed restaurants did not provide food allergy training for their staffs, and the training provided often did not cover important information such as what to do if a customer has an allergic reaction.”*				+
Wen and Kwon (2017) (15)	USA		K:	*“Restaurant servers lacked knowledge about common food allergens, differences between food allergies and food intolerances, and government regulations related to food allergy prevention and management.”*	+
			A:	*“Most restaurant servers perceived that initiating communication and preventing food allergy reactions were mostly the responsibilities of customers with food allergies.”*	+
			P:	*“Few restaurant servers who participated in this research would initiate conversations with customers about their food allergies or dietary restrictions.”*	+
Lee and Barker (2017) (41)	USA		K:	*“This study revealed gaps in food allergy knowledge among foodservice employees.”*	+
			A:		−
			P:	*“Most employees had not received food allergy training despite managers reporting that training had been provided.”*	+
Lee and Sozen (2018) (42)	USA		K:	*“Both groups were able to identify certain symptoms of allergic reactions to food but lacked knowledge of allergen-handling practices.”*	+
			A:	*“Both groups of respondents felt that customers with food allergies should inform them about their conditions…”*	+
			P:	*“Less than 30 percent of the respondents in this study stated that food allergens were noted on their menus.”*	+
					McAdams et al. (2018) (13)	Canada	Food and Drugs Act and the Safe Foods for Canadians Act	K:	*“Most participants were knowledgeable about food allergies.”*	++
A:	*“Respondents expressed highly positive attitudes toward giving special attention and care when preparing and serving dishes to customers with food allergies.”*	+++					
P:	*“There was a general lack of access to important food allergy risk management resources and training.”*	+					
South America					
Ajala et al. (2010) (12)	Brazil	Agência Nacional de Vigilância Sanitária (ANVISA)	K:	*“There is no real concern by these establishments in preparing safe meals in terms of food allergies.”*	+
			A:		−
			P:	*“There is no real concern by these establishments in preparing safe meals in terms of food allergies.”*	+
Western Europe					
Bailey et al. (2011) (33)	United Kingdom	Food Information to Consumers (FIC) Regulation 1169/2011	K:	*“This study demonstrates some worrying gaps in restaurant staff’s knowledge of food allergy.”*	+
			A:	*“Staff with high comfort and low knowledge are potentially dangerous, as they may convey an exaggerated sense of competence to their customers.”*	+
			P:	*“This research has provided evidence that restaurant staff are under-trained and under-informed about food allergy.”*	+
Common et al. (2013) (43)	United Kingdom		K:	*“This study demonstrates some worrying gaps in restaurant staff’s knowledge of food allergy.”*	+
			A:	*“Despite alarming gaps in knowledge all staff expressed ‘comfort’ in providing a safe meal to food allergic customers.”*	+
			P:	*“Very few staff had received specific formal training on how to prepare and serve food for their allergic customers.”*	+
Soon (2018) (34)	United Kingdom		K:	*“Although most takeaways’ staff demonstrated good level of food allergy knowledge, there still exist some misunderstanding of food allergens.”*	++
			A:	*“Experienced staff and managers/owners also reported more positive attitude towards food allergen management practices compared to new staff and kitchen crew.”*	++
			P:	*“Clear communication between front service staff, customers and kitchen crew are important to ensure correct allergen-free meals are prepared and delivered.”*	++
Lessa et al. (2016) (44)	Spain	Regulations (EC) No 1169/2011	K:	*“Even though the survey showed that food handlers and general public had some knowledge on the issue, a major proportion of respondents do not believe the meals produced in their restaurants are safe in terms of food allergies.”*	+
			A:	*“A major proportion of respondents do not believe the meals produced in their restaurants are safe in terms of food allergies, understandably, when one considers that chefs have varying knowledge of food allergies.”*	+
			P:	*“Restaurants should work toward providing not only food safety training as it relates to preventing microbial contamination but also provide training specific to food allergies.”*	+
Loerbroks et al. (2019) (28)	Germany		K:	*“Our study suggests that food allergy knowledge levels […] required improvement among restaurant staff.”*	+
			A:	*“Attitudes were generally positive – except for attitudes towards serving customers with food allergies and the validity of customer-reports of food allergies.”*	+
			P:		−
Eastern Europe					
Wojtyniak et al. (2013) (45)	Poland	Regulations (EC) No 1169/2011	K:	*“The knowledge about food allergy among restaurant workers is insufficient”*	+
			A:		−
			P:		−
Sogut et al. (2015) (46)	Turkey		K:	*“There are gaps in the food allergy knowledge of restaurant personnel.”*	+
			A:	*“Most of the respondents expressed that they were comfortable serving a meal to customers with food allergy.”*	+
			P:	*“The training of restaurant personnel in food allergy may be useful.”*	+
Jianu and Golet (2019) (47)	Romania		K:	*“The results also revealed important gaps in knowledge of food allergies.”*	+
			A:		−
			P:	*“Workers […] use best practice some of the time, suggesting that existing training programs […] are inadequate.”*	++
Bujaka and Riekstina-Dolge (2019) (48)	Latvia		K:	*“The respondents’ knowledge could be evaluated as poor.”*	+
			A:	*“There are further studies needed in order to explain reasons for the careless attitude […] toward the provision of allergen information.”*	+
			P:	*“Indication ‘Ask the waiter about allergens’ cannot be sufficient for the provision of allergen information.”*	+
Eren et al. (2021) (29)	Turkey		K:	*“The findings of this study indicated that chefs had moderate food allergy knowledge.”*	++
			A:	*“The participants indicated a high level of willingness to learn more about food allergies.”*	+++
			P:	*“The findings […] indicated that chefs […] had positive attitudes and practices for food allergy.”*	+++
Middle East					
Nasseredine et al. (2021) (14)	Lebanon	Ministry of public health	K:	*“The results of this study show that […] many had limited knowledge […] related to food allergies.”*	+
			A:	*“The results of this study revealed that all food service workers and managers had positive attitudes towards serving ‘special customers’.”*	+++
			P:	*“Many had […] malpractices related to food allergies.”*	+
Abdullah A. Khafagy et al., (2022) (49)	Saudi Arabia	Saudi Food and Drug Authority (SFDA)	K:	*“There is a general lack of awareness of CD, and most restaurants lack gluten-free options.”*	+
			P:	*“This indicates a lack of awareness among restaurants about an important disease that affects our population.”*	+
			A	*“Only 17.5% of the participating restaurants serve gluten-free meal options […]”*	+
Ghadi A. Alkhalaf et al. (2024) (35)	Saudi Arabia		K:	*“The study revealed a significant gap in their understanding of FA management and allergen handling practices.”*	+
			A:	*“Most staff exhibited positive attitudes toward managing food allergies.”*	+++
			P	*“Only 14% of restaurants provided allergen information on their menus.”*	+
Asia					
Shafie and Azman (2015) (17)	Malaysia	No regulations described	K:	*“Many food handlers misunderstand key information about food allergy prevention, including cross contamination.”*	+
			A:	*“Only a small proportion of them have excellent food allergy knowledge, practice and attitudes.”*	++
			P:	*“Customers with food allergies will continue to be put at risk by the restaurant staff’s poor understanding of food allergen risks […]”*	+
			Tan A. L. et al. (2023) (36)	Malaysia	K:	*“Knowledge of food allergies among food handlers in Malaysia was relatively poor.”*	+
A:	*“Managers and servers had positive attitude towards providing accurate information.”*				+++
P:	*“Respondents were not aware of the need to use separate equipment while handling allergen-free food.”*				+
Oceania					
Wham and Sharma (2014) (50)	New Zealand	The Australia New Zealand Food Standards Code (the Code)	K:	*“Our survey demonstrated that there were deficits in knowledge to identify and manage allergen risks.”*	+
			A:	*“A false sense of security can arise.”*	+
			P:	*“Few establishments had plans in place to manage food allergies or to manage an emergency.”*	+

The coding process was guided by a structured interpretive rubric developed for each KAP dimension. This rubric defined performance levels and was based on typical author language used in the original articles. The full rubric is available in Appendix 1 as a methodological reference.

## 3 Results

The sociodemographic characteristics of the 397 foodservice workers surveyed in Santiago, Chile, are presented in Table 2A. Participants ranged from 18 to 60 years of age, with more than half reporting technical or university education. Positions are identified in the table, but results were analyzed globally. Characteristics of the food services appear in Table 2B.

**Table 2:** Sociodemographic data of respondents.

		*n*	%
(A) Characteristics of the staff surveyed			
Sex	Female	201	50.6
Age	<30	233	58.7
	30–40	106	26.7
	>40	58	14.6
Nationality	Chilean	270	68.0
Education	High school or less	112	28.2
	Technical education	84	21.2
	High school or higher education	201	50.6
Professional experience	<1 year	45	11.3
	1–10 years	263	66.2
	>10 years	89	22.4
Position	Waiters	233	58.7
	Management	125	31.5
	Kitchen staff	39	9.8
(A) Workers’ characteristics			
Category	Independent	285	71.8
	Chain or franchise	99	24.9
	Do not know/no answer	13	3.3
Classification	Restaurant	191	48.1
	Cafeteria/bar/pub/hotel	139	35.0
	Fast food or take-away establishment	64	16.1
	Do not know/no answer	3	0.8
Experience	<1 year	54	13.6
	1–10 years	185	46.6
	>10 years	76	19.1
	Do not know/no answer	82	20.7
		**397**	**100**

### 3.1 Knowledge, attitudes, and practices

Eighty-seven percent of responders declared that they had not received training/information about food allergy, celiac disease, or other conditions treated with restrictive diets. Of those that did receive some training, this lasted less than 16 h in 72.4%. In case of witnessing that a dish is being contaminated during preparation, 85.1% thought that the dish had to be changed for a new one. 97.5% declared that the most important behavior was to call for medical help; however, none reported the existence of a formal emergency protocol in their establishment. The questionnaire specifically asked about structured plans rather than general actions, which may explain this discrepancy. 57.9% of the food services personnel assessed stated that persons with special dietary needs were well accepted where he/she worked. 93.7% declared that “it is the responsibility of the client to inform about his/her condition.” Notably, 45.5% of participants reported they had not served clients requesting special diets during the past year, whereas 21.2% reported receiving such clients one to three times per week. Only six waiters (2.6%) reported personal experience during their working time with food reactions in allergic or celiac customers.

To analyze knowledge, attitudes, and practices, two cutoffs were used, defining a “good” level when 50% or 80% of answers were correct. When using the 80% criterion, nearly all analyses yielded an “insufficient” level, so the following results are presented using the 50% criterion. Only 15% scored “good” in knowledge, and approximately 1% had good attitudes and practices. The evaluation of whether some variables influence knowledge, attitudes, and practices showed that, in general, participants were not aware of the relevance of the infrastructure where they prepare and serve dishes that require the elimination of some components. Better knowledge was associated with higher education, longer time (experience) working in a food service, having a manager/supervisor position, cafeterias (versus large restaurants), and long-standing restaurants (Tables 3A,B). Attitudes were related to Chilean nationality, belonging to large chain restaurants, and offering longer menus. Practices were poorer in large chain restaurants, in restaurants offering longer menus, and were better in long-lived restaurants.

**Table 3:** Knowledge, attitudes, and practices in the staff assessed, using 50% correct responses as the definition of “good” answer.

	50%		
	K	A	P
(A) Staff characteristics			
Age	0.8808	0.645	0.2659
Sex	0.5262	0.9207	0.5203
Nationality	0.2121	**0.0348**	**0.0001**
Education	**0.0057**	0.3878	0.5219
Professional experience	**0.0314**	0.6144	0.4882
Position	**<0.0001**	0.356	0.5164
(B) Food service characteristics			
Type of service where he/she last worked	**<0.0001**	0.1835	**0.0024**
Type of food service	0.1119	**0.0023**	**0.0465**
Food service classification	0.0641	0.0588	0.1556
Type of menu/carte/meal offered	**0.027**	**0.0082**	**0.0116**
Food service operating time	**0.0319**	0.6355	0.2434

Bold values indicate statistically significant associations.

### 3.2 Factors associated with knowledge, attitudes, and practices (KAP)

The Tables 3A,B also summarizes the association between individual and food service variables with accepted performance in knowledge, attitudes, and practices (KAP), using a 50% correct response threshold. Significant associations were observed between educational level, professional experience, and job position within the food service and knowledge scores. In contrast, attitudes and practices showed less variability across the evaluated factors, although differences were found according to nationality, type of service, and menu characteristics.

The statements included in Table 1 correspond to literal excerpts reported by the original authors in the results, discussion, or conclusion sections of each article. The assigned performance scores (from + to +++) reflect a structured qualitative coding process conducted independently by four reviewers. This classification was based on the criteria specified in the rubric presented in Appendix 1. It is intended to support international comparison and interpretive benchmarking and should not be considered a meta-analysis or formal statistical synthesis.

A major challenge identified during the international comparison process was the absence of a standardized definition of what constitutes a “correct response” or an “acceptable level” in KAP assessments. Studies applied diverse thresholds such as means, medians, or locally defined cutoffs, which complicates direct comparison. This methodological heterogeneity limits the generalizability of findings and highlights the need for globally harmonized metrics to support allergen risk governance. A unified evaluative framework would improve comparability across studies and contribute to the design of more effective global food safety policies.

## 4 Discussion

To our knowledge, this study is the first exploratory benchmarking of knowledge, attitudes, and practices (KAP) on food allergies (FA) and celiac disease (CD) among foodservice workers, presenting Chile as a case study. The findings reflect an overall pattern of poor or inconsistent performance across all three dimensions, consistent with reports from multiple international studies. This situation points to the persistence of structural gaps that are not solely attributed to individual characteristics of the consumer but also operational and regulatory factors. In this study, structural vulnerabilities are understood as systemic risks beyond individual staff attributes, including employment precarity (e.g., foreign-born workers), lack of standardized training, and absence of institutional protocols.

We will now discuss the comparative analysis with the normative and operational landscape in other countries, based on the international synthesis used as an interpretive framework for the Chilean case study (Table 3). The results emphasize the systemic weaknesses of risk management practices, as well as insufficient regulatory supervision, placing vulnerable consumers at avoidable risk when dining out. In Chile, allergen disclosure is not required in restaurants, and none of the surveyed establishments reported providing such information. In contrast, jurisdictions such as the European Union and certain U.S. states mandate disclosure or training, with studies reporting partial but measurable improvements, including increased written information and improved recognition of anaphylaxis. While direct quantitative comparisons were not possible in this exploratory study, these contrasts highlight Chile’s regulatory lag relative to contexts where disclosure frameworks exist. This result should be interpreted with caution, as it reflects a lack of institutional preparedness rather than a complete inability of staff to respond. The finding underscores the absence of standardized emergency planning in Chilean foodservice, despite the general willingness of staff to seek help. This study goes beyond the well-established observation that regulation without enforcement is ineffective. Our findings confirm that allergen and gluten safety in Chilean foodservice is not institutionally managed but shaped primarily by informal, individual factors. This conclusion is strongly supported by our data. For example, 93.7% of participants placed responsibility on the customer rather than the establishment. Moreover, most reported never having received formal training, and subgroup differences were associated more with individual attributes (education, nationality, establishment type) than with standardized institutional protocols. This pattern underscores the urgent need to strengthen formal training and regulatory frameworks, as current practices remain driven more by personal judgment than by institutional preparedness. Our results should be interpreted as an exploratory diagnostic. They highlight the need for pilot projects and broader surveys to build the evidence base for a phased national strategy, aligned with Codex Alimentarius and WHO/FAO recommendations, before considering nationwide implementation. In conclusion, this study not only provides the first empirical evidence on foodservice KAP in Chile but also introduces a methodological innovation by integrating local data with an international benchmarking matrix through ordinal coding. This framework can be replicated in other emerging economies to strengthen global comparisons. Our findings confirm that allergen disclosure is entirely absent in Chilean restaurants. By contrast, evidence from the European Union and the United States shows that mandatory disclosure or training can yield measurable safety benefits. These examples reinforce the urgency of developing regulatory frameworks in Chile, while recognizing that broader studies are needed to evaluate their effectiveness locally. Only 15% of respondents demonstrated acceptable knowledge, and barely 1% met basic standards for attitudes or practices, an alarming result given the increasing need for dietary restrictions in urban populations (7). Some other findings were also in line with results obtained by other authors. Significant associations revealed that higher education (*p* = 0.0057) and job seniority (*p* = 0.0314) were linked to better knowledge scores (13, 23). The strongest predictor was job position (*p* < 0.0001), with managers scoring substantially higher than waiters (27, 28). Foreign-born workers showed significantly poorer practices (*p* = 0.0001), likely due to language barriers or limited access to formal training (14, 17). Restaurants with complex menus and chain affiliations also showed lower practice scores (28, 29). These results are also in line with the literatures on the risks of cross-contact and poor allergen communication (9, 16). As in other countries, Chile lacks a legal obligation of allergen/gluten disclosure in restaurants. The RSA DS 977/96 (21) mandates hygienic food handling training but not allergen/gluten specific training or labeling.

This contrasts with more advanced regulatory frameworks. In the United States, while the Food Allergen Labeling and Consumer Protection Act (FALCPA) regulates allergens in packaged foods, it does not comprehensively apply to foodservice establishments (30), and only a few states, such as Massachusetts, have enacted specific training legislation (16, 31). In Canada, national legislation appears to have favored better knowledge levels among foodservice staff (13). In Latin America, regulation is generally limited or absent. Brazil’s RDC 26/2015 applies only to prepackaged foods and excludes restaurant services from its scope (12, 32).

In Europe, Regulation (EU) 1169/2011 requires allergen disclosure for both packaged and unpackaged foods; however, the studies available show that outcomes vary by country. In the United Kingdom these show mixed knowledge levels among staff (33, 34), while countries like Germany and Turkey report significant deficiencies (28, 29). Similar situations have been observed in parts of the Middle East and Asia, where regulations are either scarce or poorly enforced, and staff often demonstrate limited knowledge despite positive attitudes toward allergen safety (14, 35, 36).

## 5 Limitations and future research

Some of the limitations of this study refer to assessing a convenience sample, which limits representativeness, and the *ad-hoc* instruments used to collect data, which, although pilot-tested, were not validated questionnaires. The questionnaire was developed *ad hoc*, as no validated instrument currently exists for assessing KAP in foodservice allergen management. Items were extracted from our prior systematic review (22), adapted from published surveys, and reviewed by experts to ensure content validity. The instrument was also piloted in two foodservice establishments including both managers and operational staff, which allowed refinement of item clarity and applicability. These steps ensured face and content validity; however, the absence of formal psychometric testing (e.g., factor analysis, internal consistency, test–retest reliability) remains a methodological limitation that may affect measurement comparability across studies. Future research should use harmonized KAP tools and consider mixed-methods approaches combining surveys with observational audits or simulated customer tests. As a result, findings from these categories highlight critical gaps but should not be considered statistically generalizable due to the very small subgroup sizes. Also, the international comparison, while systematic, is not a meta-analysis. No eligible studies were identified from Africa, which constitutes a geographic gap in the available literature. A formal review of policy effectiveness across regions would further strengthen the benchmarking model proposed here.

## 6 Recommendations and conclusion

Based on this study and the literature analysis, we propose some recommendations: (i) follow mandatory allergen/gluten modules in certification (31, 37), (ii) standardized menu labelling (18), (iii) risk-based inspections (13), (iv) restaurant certification programs (38) and (v) The feasibility of these recommendations in Chile faces certain barriers, such as costs for small businesses, industry resistance, and limited enforcement capacity. However, relevant facilitators exist: the RSA already mandates general food safety training that could be expanded to allergens, public health priorities reinforce consumer transparency, and pilot programs could serve as scalable models. Considering these factors will help ensure that policies are realistically implementable in the foodservice sector.

We conclude that there are critical regulatory and operational gaps in food allergy and celiac disease management. In the absence of binding laws, most restaurants rely on personal judgment and poor knowledge, exposing consumers to preventable risks.

Chile is not alone in this. Other upper-middle-income countries face similar challenges. However, international evidence shows that improvement is feasible. Where regulation, training, enforcement, and consumer transparency co-exist, food safety outcomes improve measurably.

Our findings provide a replicable diagnostic framework for emerging economies aiming to enhance allergen governance. A national strategy—rooted in Codex Alimentarius and WHO/FAO guidelines—is not only advisable but imperative to uphold the right to safe food for all citizens, regardless of dietary restrictions.

## Data Availability

The raw data supporting the conclusions of this article will be made available by the authors, without undue reservation.
