# Correction: Benchmarking knowledge, attitudes and practices on food allergies and celiac disease among food service staff: exploratory findings and policy gaps

**DOI:** 10.3389/fnut.2025.1762234

**Published:** 2026-01-12

**Authors:** Ximena Figueroa-Gómez, Juan Manuel Rodríguez, María-Jesús Oliveras-López, Marcelo Poyanco, Herminia López, Fernando Martínez-Martínez, Magdalena Araya

**Affiliations:** 1 Department of Pharmacy and Pharmaceutical Technology, Faculty of Pharmacy, University of Granada, Granada, Spain; 2 Human Nutrition Unit, Institute of Nutrition and Food Technology (INTA), University of Chile, Santiago, Chile; 3 Institute of Nutrition and Food Technology (INTA), University of Chile, Santiago, Chile; 4 Department of Molecular Biology and Biochemical Engineering, University Pablo de Olavide, Seville, Spain; 5 Faculty of Economic and Administrative Sciences, University of Valparaíso, Viña del Mar, Región de Valparaíso, Chile; 6 Department of Nutrition and Bromatology, Faculty of Pharmacy, University of Granada, Granada, Spain; 7 Department of Pharmacy and Pharmaceutical Technology, Faculty of Pharmacy, University of Granada, Granada, Spain

**Keywords:** food service, food allergy, celiac disease, knowledge, attitudes, practices

The full reference for Reference 6 was erroneously written in the **Reference List** as published. It was written as, “6. Figueroa-Gómez X, Oliveras-López MJ, Rodríguez Silva JM, Poyanco M, López H, Araya M. Experiences and perceptions of people with celiac disease, food allergies and food intolerance when dining out. *Front Nutr*. (2024) 11:1–8. doi: 10.1016/j.jaci.2005.12.1303. It should have been written as, “6. Figueroa-Gómez X, Oliveras-López MJ, Rodríguez Silva JM, Poyanco M, López H, Araya M. Experiences and perceptions of people with celiac disease, food allergies and food intolerance when dining out. *Front Nutr*. (2024) 11:1321360. doi: 10.3389/fnut.2024.1321360

The original version of this article has been updated.

