# Supplementary material for: Benchmarking knowledge, attitudes and practices on food allergies and celiac disease among food service staff: exploratory findings and policy gaps
Source: Front Nutr. 2025 Oct 23;12:1644906. doi: 10.3389/fnut.2025.1644906 (PMC12588833; doi:10.3389/fnut.2025.1644906)
Supplement: Supplementary file 1 [file Table_1.docx]

**Appendix 1**

**A.1 Coding Rubric – Dimension: Knowledge (K)**

| **Category** | **General description of knowledge level** | **Typical author language indicators** |
| --- | --- | --- |
| +++ (good) | Staff possesses adequate or high-level knowledge about food allergens. | “High level of knowledge”, “well-informed”, “good understanding”, “appropriate knowledge”, “aware” |
| ++ (moderate) | Intermediate or acceptable knowledge, with gaps or occasional errors. | “Moderate knowledge”, “some gaps”, “acceptable level”, “partial understanding”, “needs improvement” |
| + (insufficient) | Deficient or incorrect knowledge; lack of basic understanding. | “Low awareness”, “poor knowledge”, “limited knowledge”, “lack of understanding”, “inadequate” |
| — (not stated) | The study does not provide specific information about knowledge. | The dimension is not explicitly addressed in results or discussion. |

**A.2 Coding Rubric – Dimension: Attitude (A)**

| **Category** | **General description of attitude level** | **Typical author language indicators** |
| --- | --- | --- |
| +++ (good) | Staff demonstrates a positive, proactive, and committed attitude toward managing food allergies. | “Very positive attitude”, “strong commitment”, “supportive”, “willing to protect allergic consumers” |
| ++ (moderate) | Generally favorable attitude, but with limited awareness or consistency. | “Moderate attitude”, “partially positive”, “some awareness”, “needs reinforcement”, “variable concern” |
| + (insufficient) | Negligent, indifferent, or insensitive attitudes toward the issue. | “Indifferent”, “lack of concern”, “poor attitude”, “blame on customer”, “not serious about allergies” |
| — (not stated) | The study does not provide specific information about attitudes. | No mention of attitudes in results, discussion, or conclusions. |

**A.3 Coding Rubric – Dimension: Practices (P)**

| **Category** | **General description of practice level** | **Typical author language indicators** |
| --- | --- | --- |
| +++ (good) | Staff implements correct, systematic practices aligned with allergen safety protocols. | “Appropriate practices”, “standard procedures followed”, “preventive protocols implemented”, “trained actions” |
| ++ (moderate) | Some appropriate practices are applied, but inconsistently or partially. | “Some practices applied”, “inconsistent implementation”, “room for improvement”, “variable adherence” |
| + (insufficient) | Inadequate, unsafe, or absent practices for managing food allergens or celiac risk. | “Unsafe practices”, “lack of procedures”, “no allergen management”, “incorrect handling”, “not trained” |
| — (not stated) | The study does not provide specific information about practices. | No mention of operational or behavioral practices in the study text. |
